# Proteome Profiles of Head Kidney and Spleen of Rainbow Trout (*Oncorhynchus Mykiss*)

**DOI:** 10.1002/pmic.201800101

**Published:** 2018-08-16

**Authors:** Gokhlesh Kumar, Karin Hummel, Ebrahim Razzazi‐Fazeli, Mansour El‐Matbouli

**Affiliations:** ^1^ Clinical Division of Fish Medicine University of Veterinary Medicine Vienna Austria; ^2^ VetCore Facility for Research / Proteomics Unit University of Veterinary Medicine Vienna Austria

**Keywords:** lymphoid organs, salmonids, trout proteomics

## Abstract

The head kidney and spleen are major lymphoid organs of the teleost fish. The authors identify proteome profiles of head kidney and spleen of rainbow trout (*Oncorhynchus mykiss*) using a shotgun proteomic approach. Gene ontology annotation of proteins is predicted using bioinformatic tools. This study represents detailed proteome profiles of head kidney and spleen of rainbow trout, with a total of 3241 and 2542 proteins identified, respectively. It is found that lymphoid organs are equipped with a variety of functional proteins related to defense, receptor, signal transduction, antioxidant, cytoskeleton, transport, binding, and metabolic processes. The identified proteome profiles will serve as a template for understanding lymphoid organ functions in salmonids and will increase the amount of spectra information of rainbow trout proteins in the public data repository PRIDE. This data can be accessed via ProteomeXchange with identifiers PXD008473 and PXD008478.

Rainbow trout (Salmonidae) is a fish species that is fast growing and important to commercial aquaculture, constituting the most widely farmed salmonid species in hatcheries in many countries. Rainbow trout constitute a major source of proteins, high levels of vitamins, and essential micronutrients.^1^ Some diseases, such as proliferative kidney disease,^2^ enteric redmouth disease,^3^ and furunculosis,^4^ are the major concerns in salmonids, where these diseases significantly impact on kidney and spleen organs of infected fish and cause high economic losses to fish farmers and fishing industries. In teleost fish, the head kidney is a haematopoietic, lymphoid, and endocrine tissue while the spleen is the secondary lymphoid organ.^5^ Proteome profiles of sperm, seminal plasma, and ovarian fluid have been identified from rainbow trout.^6^, ^7^, ^8^ There is limited knowledge about detailed proteome of rainbow trout lymphoid organs. Only differential protein expression profiles in the spleen of rainbow trout have been examined to investigate the immune mechanisms against *Aeromonas salmonicida* infection.^9^ To understand lymphoid organs functions and disease, it is important to define the molecular constituents of the various compartments of the head kidney and spleen proteome profiles of rainbow trout.

The aim of the present study was to identify whole proteome profiles of head kidney and spleen of rainbow trout using a gel‐free, label‐free shotgun proteomics approach. This study represents descriptive and comparative proteomic profiles of lymphoid organs of rainbow trout.

Specific pathogen‐free rainbow trout (mean length 15 ± 1 cm) were maintained in recirculating dechlorinated water at 19 ± 1 °C with constant aeration. Fish were fed at a rate of 1% biomass with commercial pelleted feed. Fish (*n* = 27, Figure 1a) were anaesthetized with MS‐222 (Sigma) and individual organs were sampled aseptically. Head kidney and spleen (Figure 1b) were washed three times with sterile phosphate‐buffered saline containing a cocktail of mammalian protease inhibitors and snap‐frozen in liquid nitrogen and stored at –80 °C. This study was approved by the institutional ethics committee of the University of Veterinary Medicine Vienna (BMWFW‐68.205/0041‐WF/V/3b/2015).

**Figure 1:**
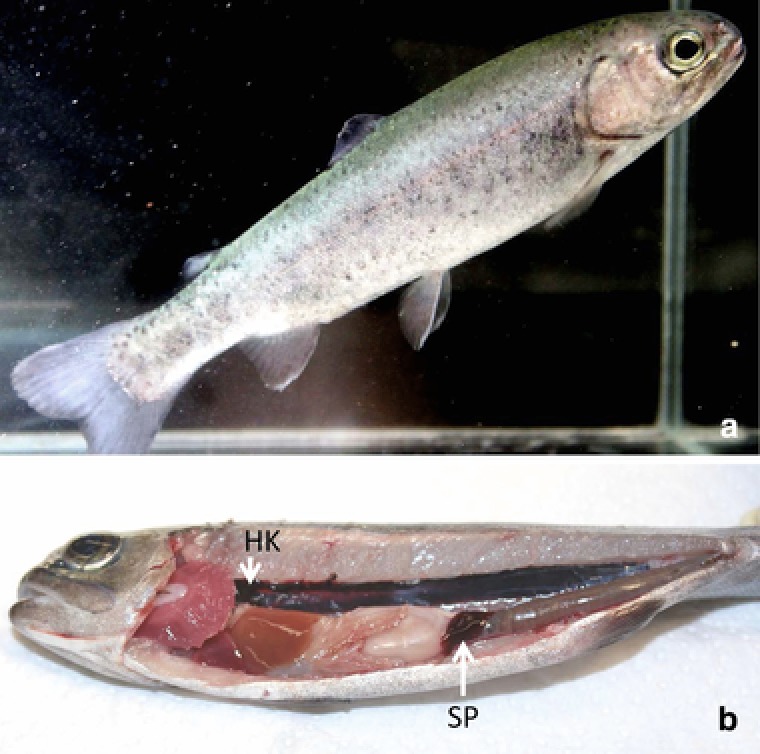
Overview of rainbow trout. a) External morphology and b) internal organs. HK, head kidney; SP, spleen.

Equal amounts of head kidney or spleen of three individual tissues were pooled to minimize the effects of individual variation and compensate for the low sample amount. In total 9 pools (i.e., *n* = 9 biological replicates) for each organ were prepared for proteomic analysis. Tissue samples were ground into a fine powder using a mortar and pestle in liquid nitrogen. Each powder sample was suspended in lysis buffer containing 7 M urea, 2 M thiourea, 4% CHAPS, 1% DTT, and mammalian protease inhibitor cocktail and sonicated. After incubation overnight at 4 °C, the samples were centrifuged at 12 000 rpm for 20 min at 4 °C. The protein yield for each sample was measured using a Pierce 660 nm Protein Assay Kit.

Each sample (30 μg) was digested with Trypsin/Lys‐C mix (Promega) according to the standard enhanced filter‐aided sample preparation protocol using Amicon Ultra 0.5 mL Ultracel 10 K centrifugal filters.^10^ Digested peptides were extracted from the filter with three changes of 50 mM ammonium bicarbonate. Sodium deoxycholate was removed by phase transfer with ethyl acetate. Afterward, peptides were dried and redissolved in 0.1% aqueous TFA.

Peptide separation was performed on an Eksigent NanoLC 425 system (Sciex) using a microflow pump module. A 5 mm YMC‐Triart C18 precolumn was applied for sample preconcentration and desalting. Sample loading and desalting were achieved using ultra‐pure LC–MS grade H_2_O with 0.1% formic acid (FA) as a mobile phase with a flow rate of 10 μL/min. The following peptide separation was performed on a 15 cm YMC‐Triart C18 column with a flow rate of 5 μL/min. The gradient started with 3% B (ACN with 0.1% FA) and increased in two steps to 25% B (68 min) and 35% (73 min). Afterward, a washing step with 80% B was performed. Total run time of the LC–MS analysis was 87 min. Mobile Phase A consisted of ultra‐pure H_2_O with 0.1% FA. For mass spectrometric analysis, the LC was directly coupled to a high resolution quadrupole time of flight mass spectrometer (Triple TOF 5600+, Sciex).

MS1 spectra were collected in the range of 400–1250 m/z for 250 ms (information dependent data acquisition). A top 40 acquisition method fragmenting the 40 most intense precursors with charge state 2–4, which exceeded 150 counts per second, was applied. MS2 spectra were collected in the range of 200–1500 m/z for 50 ms with dynamic exclusion of precursors from reselection for 13 s. The HPLC system was operated by Eksigent Control Software version 4.2 (Sciex) and the MS by Analyst Software 1.7.1 (Sciex). Database searches were done in ProteinPilot Software version 5.0 (Sciex) using a RefSeq database containing rainbow trout proteins (taxonomy 8022) as well as a common repository of adventitious proteins, downloaded from, http://www.thegpm.org/crap/index.html. Raw files were preprocessed in order to create peak lists. These were used for database search applying the Paragon Algorithm.^11^ It creates several sequence tags for each peptide MS/MS spectrum. These are then mapped to the protein database and rated how likely each one is to be correct as so‐called sequence temperature values. Together with an internal list of probabilities for modifications, the database search was performed using the following criteria: mass tolerance in MS mode was set with 0.05 and 0.1 Da in MS/MS mode for the rapid recalibration search, and 0.0011 Da in MS and 0.01 Da in MS/MS mode for the final search. Following search parameters were specified additionally to the common parameters automatically set by the software: trypsin digestion, cysteine alkylation with iodoacetamide, and rapid ID. After the initial database search, the ProGroup Algorithm (AB SCIEX) was applied for confident assignment of the identified peptides to protein groups. Redundant proteins or homologous proteins are combined in protein groups as so‐called “ambiguous proteins”. Proteins with evidence of unique peptides are listed as distinct protein hits. So the ProGroup Algorithm helps to avoid false over‐estimation of protein ID numbers. As a last step, false discovery rate (FDR) analysis was performed using the integrated tools in ProteinPilot. FDR was set to <1% on protein as well as on peptide level.

Protein identifications are based on a minimum of two peptides identified with probabilities *>*95%. To obtain GO annotation for biological processes and molecular functions, we used the STRAP software,^12^ UniProtKB database, and gene ontology consortium (http://www.geneontology.org/). Venn diagrams were used to show the differences between protein lists originating from head kidney and spleen of rainbow trout.

A total of 3241 proteins in the head kidney (Table S1, Supporting Information) and 2542 proteins (Table S2, Supporting Information) in spleen were identified using the label‐free shotgun proteomic approach. The number of kidney proteins is consistent with that reported in other fish species: 385 proteins were identified in the kidney from zebrafish^13^; 4009 proteins in the kidney from Atlantic salmon.^14^ However, the number of spleen proteins (2542 proteins) was much higher in our study than reported by Long et al,^9^ where they found 1447 proteins in the spleen of rainbow trout in a study applying iTRAQ labeling for quantification. The overall list of identified proteins, protein score, confident peptides, sequence coverage, and ambiguous accessions to each protein is provided in Tables S1 and S2, Supporting Information. These proteins were associated with defense, cell adhesion, transferase, hydrolase, transcription, enzyme modulator, cytoskeleton, and oxidoreductase. The identified important proteins were nicotinamide adenine dinucleotide (NADPH) oxidase cytosolic proteins (p40phox and p67phox), superoxide dismutase [Cu‐Zu], BolA‐like protein 2, annexin, transgelin, peptidyl‐prolyl cis‐trans isomerase, transaldolase, fibroleukin, and death‐associated protein‐like 1‐B. These proteins play a role in phagocytosis, metal ion binding, iron‐sulfur (Fe‐S) cluster assembly, calcium ion binding, protein folding, pentose‐phosphate pathway, neutrophil degranulation, and apoptotic signaling pathway, respectively. Some of these protein‐coding genes were functionally characterized in fish organs and their gene expression is modulated in response to pathogens.^15^, ^16^, ^17^, ^18^

Based on biological processes, the identified head kidney and spleen proteome datasets were classified into several groups (Figure 2). The groups with the highest number of proteins were involved in cellular (29%), metabolic (20%) processes along with immune system (1%), response to stimulus (9%), developmental process (7%), and localization (7%). This high number of proteins involved in cellular and metabolic processes is consistent with that reported in other fish species proteome datasets: cellular process (49%) and metabolic process (9%) were identified in the kidney proteome from zebrafish^13^; cellular process (15%) and metabolic process (13%) in the kidney proteome from Atlantic salmon.^14^ Under the category of immune system process, proteins were categorized into B cell‐mediated immunity, complement activation, and response to interferon‐gamma. This strongly suggests that rainbow trout's lymphoid organs have protective immune systems against pathogens and stress responses. Furthermore, stimulus response proteins were categorized into behavior, cellular defense response, immune response, and response to external/endogenous stimulus, abiotic and biotic stimulus, and toxic substance.

**Figure 2:**
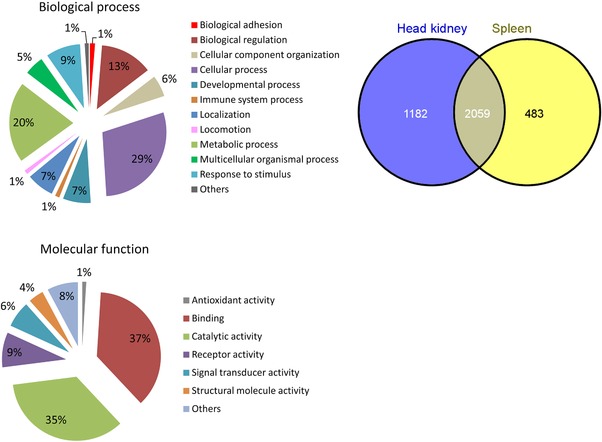
Pie chart distribution of head kidney proteome dataset of rainbow trout. The classification of the protein set was performed according to the GO terms “biological process” and “molecular function.” Venn diagram shows the number of unique or shared proteins of head kidney and spleen.

Analysis of molecular functions of identified proteome datasets showed that most of the proteins were classified as having binding (37%) and catalytic activity (35%) along with antioxidant (1%), receptor (9%), signal transducer (6%), structural molecule (4%), and other (8%) activities (Figure 2). Similar results were previously reported in other fish species, where most of the proteins were found associated with binding (50%) and catalytic activity (33%) in Zebrafish and Atlantic salmon kidney proteome datasets.^13^, ^14^ Binding activity proteins were categorized into antigen binding, calcium ion binding, carbohydrate binding, chromatin binding, lipid binding, nucleic acid binding, and protein binding. Receptor activity proteins were categorized into G‐protein coupled receptor activity, GABA receptor activity, acetylcholine receptor activity, cytokine receptor activity, tumor necrosis factor receptor activity, glutamate receptor activity, receptor inhibitor activity, and transmembrane protein serine/threonine/tyrosine kinase activity. From this perspective, identified lymphoid proteins with specific biological process and molecular function provide interesting candidates for future more in‐depth studies in trout.

As can be seen in Venn diagram (Figure 2), 55.3% proteins (2059 proteins) were commonly identified in both lymphoid organs. The presence of common proteins in head kidney and spleen suggest that these proteins are involved in the development of organs, blood immune parameters, cell growth, and protection of fish. This indicates that housekeeping genes necessary to support all cells/tissues are likely a major component of the common protein repertoire in head kidney and spleen. Proteins involved in immune system process and response to stimulus were complement C3 and C9, transporter associated with antigen processing (TAP), apoptosis‐associated speck‐like protein containing a caspase recruitment domain (ASC‐CARD), precerebellin‐like protein, lipopolysaccharide binding protein (LBP), and bactericidal permeability‐increasing protein (BPI). Components, C3 and C9, are proteins of the complement system that play a key role in host defense against infection.^19^, ^20^ A precerebellin‐like protein is suggested as a part of the acute phase response in rainbow trout.^21^ TAP is a transmembrane glycoprotein that forms a functional bridge between the transporter associated with antigen processing and the MHC class I receptor.^22^ ASC is an adaptor protein that plays a key role in PYRIN and CARD‐dependent pathways. ASC also plays a central role in multiple inflammasome protein complexes that mediate inflammation and host defense.^23^ LBP and BPI play a significant role in transducing cellular signals, and function as essential molecules for protection from bacterial invasion.^24^ Additionally, important antioxidant proteins were lysozyme C II, glutathione peroxidase, and thioredoxin, which are involved in host defense and reactive oxygen species to protect the fish from oxidative damage. All these protein‐coding genes have been characterized in rainbow trout and their expression levels have been induced in different organs of fish in response to immunomodulators and pathogens.^21^, ^22^, ^23^, ^25^, ^26^

In conclusion, this study provides in‐depth proteome of head kidney and spleen of rainbow trout. We found that lymphoid organs of rainbow trout are equipped with functionally diverse proteins related to immune system, response to stimulus, and other cellular processes. Proteome profiles achieved in this study will be useful for comparing and understanding organs biology and diseases, and will serve as an initial framework for lymphoid organs. The proteome dataset will increase the amount of information of rainbow trout proteins in the public data repository PRIDE. The improved knowledge of fish proteins will be of value to fish biologists and aquaculture research. It will open the way for more focused studies on their functions and possible protein interactions to analysis of signal transduction and proteome pathways as well as of carbohydrate, nucleotide, amino acid, and energy metabolism.

The MS proteomics data have been deposited to the ProteomeXchange Consortium (http://www.proteomexchange.org/) via the PRIDE partner repository^27^ with the dataset identifiers PXD008473 and PXD008478.

## Conflict of Interest

The authors declare no conflict of interest.

## Supporting information

Supporting information

Supporting information
